# Impact of an unprecedented marine heatwave on extremely hot summer over Northern Japan in 2023

**DOI:** 10.1038/s41598-024-65291-y

**Published:** 2024-07-19

**Authors:** Hirotaka Sato, Kazuto Takemura, Akira Ito, Takafumi Umeda, Shuhei Maeda, Youichi Tanimoto, Masami Nonaka, Hisashi Nakamura

**Affiliations:** 1 Japan Meteorological Agency, 3-6-9 Toranomon, Minato City, Tokyo 105–8431 Japan; 2 Meteorological Research Institute, Japan Meteorological Agency, Tsukuba, Japan; 3 Faculty of Environmental Earth Science, Hokkaido University, Sapporo, Japan; 4 Application Laboratory, Japan Agency for Marine-Earth Science and Technology, Yokohama, Japan; 5 Research Center for Advanced Science and Technology, University of Tokyo, Tokyo, Japan

**Keywords:** Atmospheric science, Ocean sciences, Natural hazards

## Abstract

Possible local influence of an extreme marine heatwave is investigated on unprecedentedly hot summer around northern Japan in 2023. Sea-surface temperatures (SSTs) and subsurface ocean temperatures around northern Japan were also unprecedentedly high in the summer. This was especially the case off the east coast of Japan, where cool Oyashio water was replaced with much warmer water due to a striking poleward meander of the Kuroshio Extension persistent from the spring. Particularly amplified near-surface air temperature anomalies and even stronger warm anomalies in the subsurface ocean suggest that the marine heatwave acted to sustain the atmospheric heatwave. Anomalous upward of latent and sensible heat fluxes from the warmed sea surface are indicative of local oceanic impact. The warm SST anomalies reduced the lower-tropospheric stratification to maintain unfavourable condition for low-level cloud formation, which in turn led to increased surface insolation for further SST warming as positive feedback. The increased moisture in the warmed lower troposphere contributed to the enhanced surface downward longwave radiation. This enhanced greenhouse effect acted not only as positive feedback on the warm SST anomalies that increased evaporation but also as a contributor for the extreme warmth over northern Japan landmass.

## Introduction

Recently marine heatwave (MHW) events^1^ have been attracting broad attention due to their devastating impacts on marine ecosystems and fisheries^2^. A MHW event is a phenomenon in which extremely high sea water temperatures at the particular time of the year relative to the climatology persist for days or months^1^. The frequency of MHWs has increased since the 1980s and will likely continue to increase further associated with the ongoing global warming^3^. Physical processes involved in MHW depends on season and regions, owing to the ocean internal dynamics, local air-sea interaction processes and large-scale atmospheric teleconnections^4^.

For summertime MHWs in the North Pacific, various processes have been proposed for their development and maintenance. A MHW event observed in the eastern North Pacific in 2019 summer, known as “Blob2.0”, resulted from the weak North Pacific Subtropical High under the local positive feedback, where reduced low-level cloud cover under lowered low-level static stability over extremely warm sea surface temperature (SST)^5^ and the enhanced insolation were involved. The importance of such low-cloud-SST feedback was also pointed out for a MHW event off Baja California that persisted from 2014 to 2015^6^.

Around East Asia, summertime MHWs can occur by atmospheric forcing. For example, Kuroda and Setou^7^ argued a summertime MHW event within the Kuroshio Extension (KE) region in 2021 developed under strong atmospheric forcing due to the enhanced and extended subtropical high predominant around Japan, accompanied by the poleward shift of the upper-tropospheric subtropical jetstream. Such large-scale atmospheric circulation anomalies leading to summertime MHWs in East Asia can be formed by atmospheric teleconnections^8,9^, including the Pacific-Japan (PJ) pattern^10,11^ associated with enhanced cumulus convection around the Philippines. Some summertime MHW events triggered by atmospheric teleconnection develop on top of deep warm anomalies that formed in the preceding winter under the influence of the El Niño teleconnection^8^. Meanwhile, Miyama et al.^12^ argued that warm anticyclonic ocean eddies detached from the KE increased MHW events in the Oyashio region east of northern Japan from 2010 to 2016. Du et al.^13^ pointed out that the low-cloud-SST feedback is operative in some MHW events in the Kuroshio-Oyashio region during recent decades. Still, it has not been clarified how a summertime MHW around East Asia can influence the overlying atmosphere, including an atmospheric heatwave.

In 2023 summer, SST and subsurface temperature around northern Japan were record high, which can be regarded as a MHW event, and Japan experienced unprecedented hot summer, especially in its northern part. Overall features and possible factors of the unprecedented heat wave over Japan have been reported by Takemura et al.^14^ They pointed out possible influence of the MHW around northern Japan on the atmosphere, but the specifics have not been clarified yet. Some previous studies^15,16^ argued that variability in the Kuroshio path can influence summertime surface air temperature (SAT) on the southern coast of Japan through modifying local moisture supply and related greenhouse effect. One may thus wonder what the origin of the MHW around northern Japan in summer 2023 was and how the MHW could affect the overlying atmosphere from a viewpoint of an emerging paradigm of active roles of the mid-latitude oceans in the climate system^17–19^, especially the oceanic frontal zones in the vicinity of Japan (e.g., Nakamura et al.^20^).

The present study presents the first analysis of how the MHW around northern Japan in summer 2023 influenced local atmospheric conditions. We aim at clarifying physical processes that could potentially involve local oceanic impacts behind the unprecedentedly hot summer. The maritime regions of our interest are the KE region and the Sea of Japan, which differ from their counterpart in the above-mentioned studies that investigated the local MHW impact onto the summer climate in Japan. Our findings will make an important contribution to better understanding of summertime air-sea interaction processes in the vicinity of the oceanic frontal zones around Japan.

## Results

### Oceanic and atmospheric conditions

**Figure 1:**
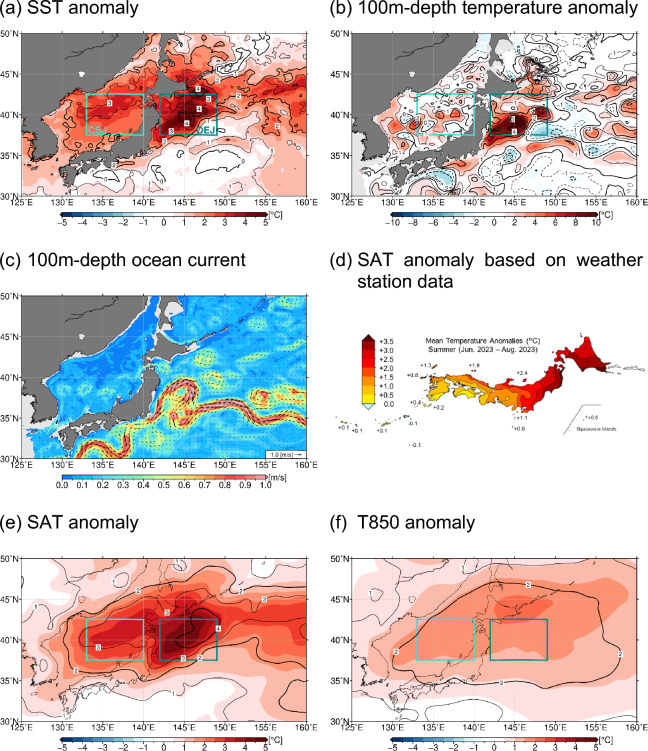
Anomalies (shaded) and normalized anomalies (contoured) by local standard deviation of interannual variations of (**a**) SST (°C), (**b**) 100 m-depth temperature (°C), (**e**) SAT (°C) and (**f**) 850-hPa temperature (°C), all averaged in the summer of 2023. Thick and thin contour intervals are 2 and 1, respectively. (**c**) 100 m-depth ocean current speed (shaded) and direction (arrows) in summer 2023 (not anomalies). (**d**) SAT (°C) anomalies based on weather station observations in summer 2023. These maps were generated with the Generic Mapping Tools (GMT) software (ver.5.4.4; https://www.generic-mapping-tools.org/download/)^42^.

**Figure 2:**
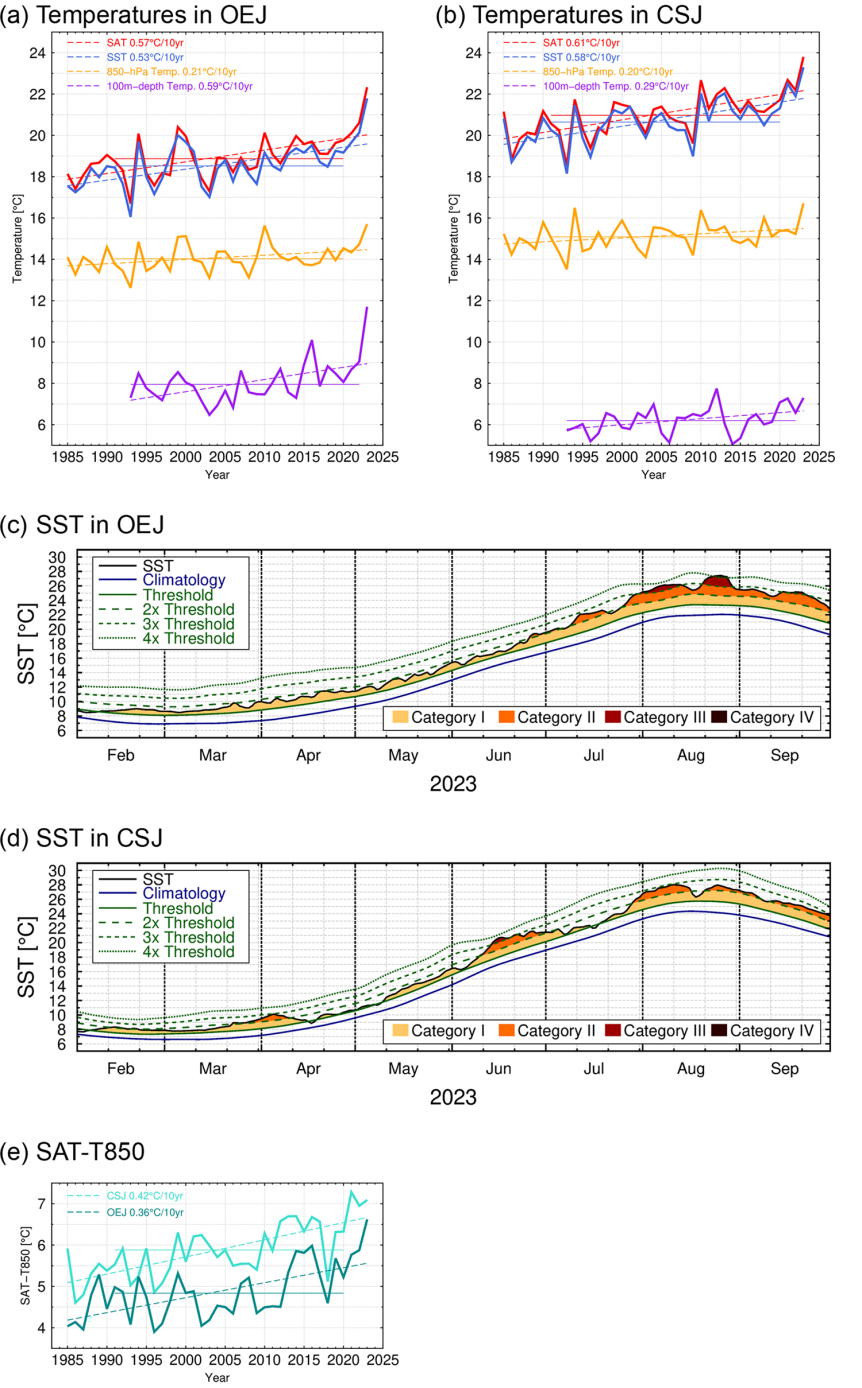
Timeseries of summer-mean SAT (red), SST (blue), 850-hPa temperature (orange) and 100 m-depth temperature (purple) averaged in (**a**) Oyashio region east of northern Japan (OEJ; 37.5°–42.5°N, 142°–149°E, green rectangular in Fig. 1) and (**b**) central portion of (**a**) in the Sea of Japan (CSJ; 37.5°–42.5°N, 133°–140°E, light blue rectangular in Fig. 1). (**e**) Timeseries of summer-mean SAT minus 850-hPa temperature for OEJ (green) and CSJ (light blue). Dashed lines indicate linear trends, shown if statistically significant at the 95% confidence level based on Student’s *t*-test. Thin horizontal lines are the climatological normal for each variable. (**c**, **d**) Timeseries of daily SST in 2023 (°C, black), climatological normal of 11-day running mean of SST (°C, blue), 90th percentile threshold of marine heatwave (°C, green solid line) for defining MHW category I (modest). The green dashed lines are twice, three times and four times of the 90th percentile anomaly from the climatological normal for defining the categories II (strong), III (severe) and IV (extreme), respectively. The categories for individual days are indicated by colours.

Overall features of oceanic and atmospheric conditions around Japan in summer (June–July–August) 2023 are summarized in Fig. 1. SST around northern Japan was substantially higher than its climatology for the 1991 ~ 2020 period (Fig. 1a). The warm SST anomalies exceeded four standard deviations (SDs) of interannual variations in the Oyashio region off the east coast of northern Japan (OEJ; green rectangular in Fig. 1a) and three SDs in the central portion of the Sea of Japan (CSJ; light blue rectangular in Fig. 1a). The area-averaged summer-mean SSTs within OEJ and CSJ (Fig. 2a,b, respectively) in 2023 were the highest during the satellite era since 1985, surpassing the anomalies in 2021, when an extensive and intense MHW event occurred in the mid-latitude western North Pacific including OEJ in July and August^7^. The SST anomalies in both CSJ and OEJ in summer 2023 were extraordinary also in long-term historical SST data since the early twentieth century, even though values in the 1940s are less reliable due to lack of observational data (Supplementary Fig. S1). The summer-mean SST anomalies in the OEJ and CSJ in 2023 were still the highest even after removing linear trends for the period of 1985 ~ 2023 (see Supplementary Fig. S2).

The extremely high SST conditions in 2023 summer around northern Japan can be regarded as unprecedented MHWs based on metrics (Fig. 2c,d, and Supplementary Figs. S3a and S3b) proposed by Hobday et al.^1,21^ (see the “Methods” section for the details). The MHW event in OEJ first reached the Category I (modest) level in early February 2023 and persisted throughout the spring and summer (Fig. 2c), during which it developed into the Category III (severe) level at the beginning of August and even into the Category IV (extreme) in late August. Meanwhile, the CSJ event occurred intermittently from February to September (Fig. 2d), but it intensified into Category III in mid-June. The summertime MHWs in both regions in 2023 were outstanding in terms of duration and intensity (Supplementary Figs. S3a,b). Their duration in summer 2023 was 92 days in OEJ and 89 days in CSJ as the most prolonged events since 1985. Furthermore, it was the first time in OEJ since 1985 that a MHW rated Category III or higher occurred. In CSJ, a MHW reaching Category IV was observed in summer 2021 but not in 2023. Nevertheless, the duration of a MHW rated Category II or higher was the longest since 1985.

**Figure 3:**
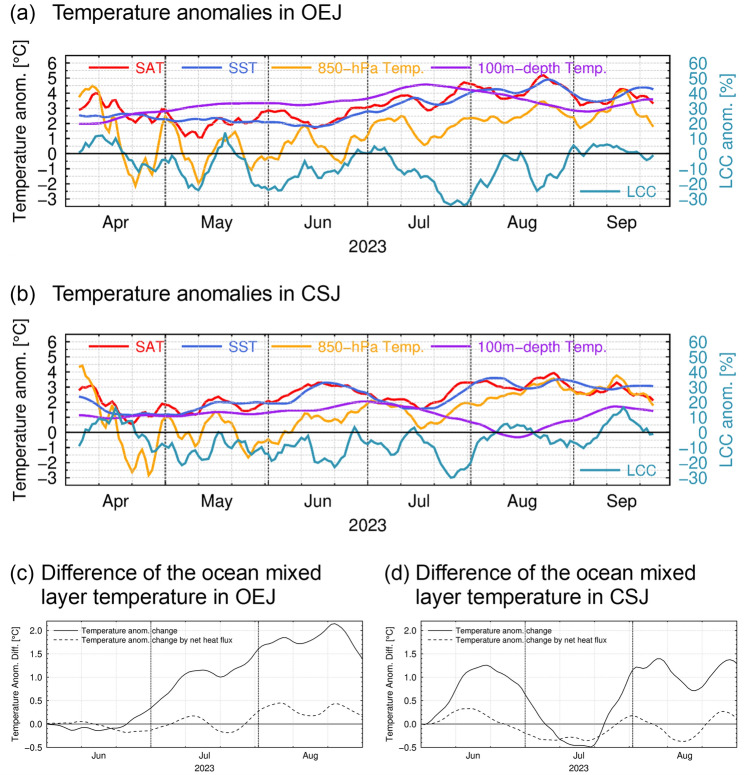
(**a**, **b**) Timeseries of 11-day running mean anomalies of SAT (°C, red), SST (°C, blue), 850-hPa temperature (°C, orange), 100 m-depth temperature (°C, purple) and low-level cloud cover (%, cyan) in 2023 for (**a**) OEJ and (**b**) CSJ. (**c**, **d**) Difference in 11-day running mean anomalies of the ocean mixed layer temperature (°C) relative to the value on 1 June 2023 (solid line) and accumulated heating by net air-sea heat flux anomalies (dashed line) after 1 June 2023 in (**c**) OEJ and (**d**) CSJ, where the anomalies are defined as deviations from 30-year averages for the 1993–2022 period in (**c**) and (**d**).

In 2023 extremely warm conditions were observed also in the subsurface ocean. For example, 100 m-depth temperature was also much higher than its climatology (Fig. 1b), reaching unprecedented levels in OEJ (Fig. 2a). The anomaly in OEJ exceeded 4 ~ 5 SDs and was still the highest even after removing a linear trend since 1993 (see Supplementary Fig. S2a). As evident in Fig. 1c, the pronounced SST and subsurface temperature anomalies observed in OEJ in 2023 summer were associated with the extreme poleward meander of the KE reaching as far north as 40°N compared to its climatological latitude around 36°N. This large KE meander had continued at latest since the preceding spring, which is one of the distinguished features of the MHW in 2023 from the other summertime MHWs around Japan as mentioned above. In fact, the warm anomalies of SST and 100 m-depth temperature had persisted in both OEJ and CSJ since 2023 spring (Fig. 3a,b). As shown in Fig. 3c,d, our crude estimate of the ocean mixed layer heat budget suggests that the warming by anomalous net air-sea heat exchanges via shortwave radiation, longwave radiation, latent heat and sensible heat fluxes (see the “Methods” section for details) was unlikely a primary factor of the maintenance of the MHWs around northern Japan in summer 2023. Rather, oceanic internal processes such as advection and entrainment seem crucial. This limited contribution of the air-sea heat flux anomalies was obtained under the assumption of the fixed ocean mixed layer depth (*h*) of 10 m, which is shallower than its observational counterpart as shown later and thus likely to overestimate the heat flux contribution. This further supports the importance of oceanic internal processes. Nevertheless, the anomalous air-sea heat exchange acted to reinforce the MHW in OEJ from late July into August, when enhanced shortwave radiation under the reduced low-level cloud cover (Fig. 3a) surpassed the counteracting effect by other heat flux anomalies (see Supplementary Fig. S4a). While 850-hPa air temperature (*T*_*850*_) was often below or around the climatology from April to July (Fig. 3a,b), anomalies of SAT, SST and 100m-depth temperature around northern Japan remained positive, suggesting that the persistent warm ocean anomalies sustained the SAT anomalies.

**Figure 4:**
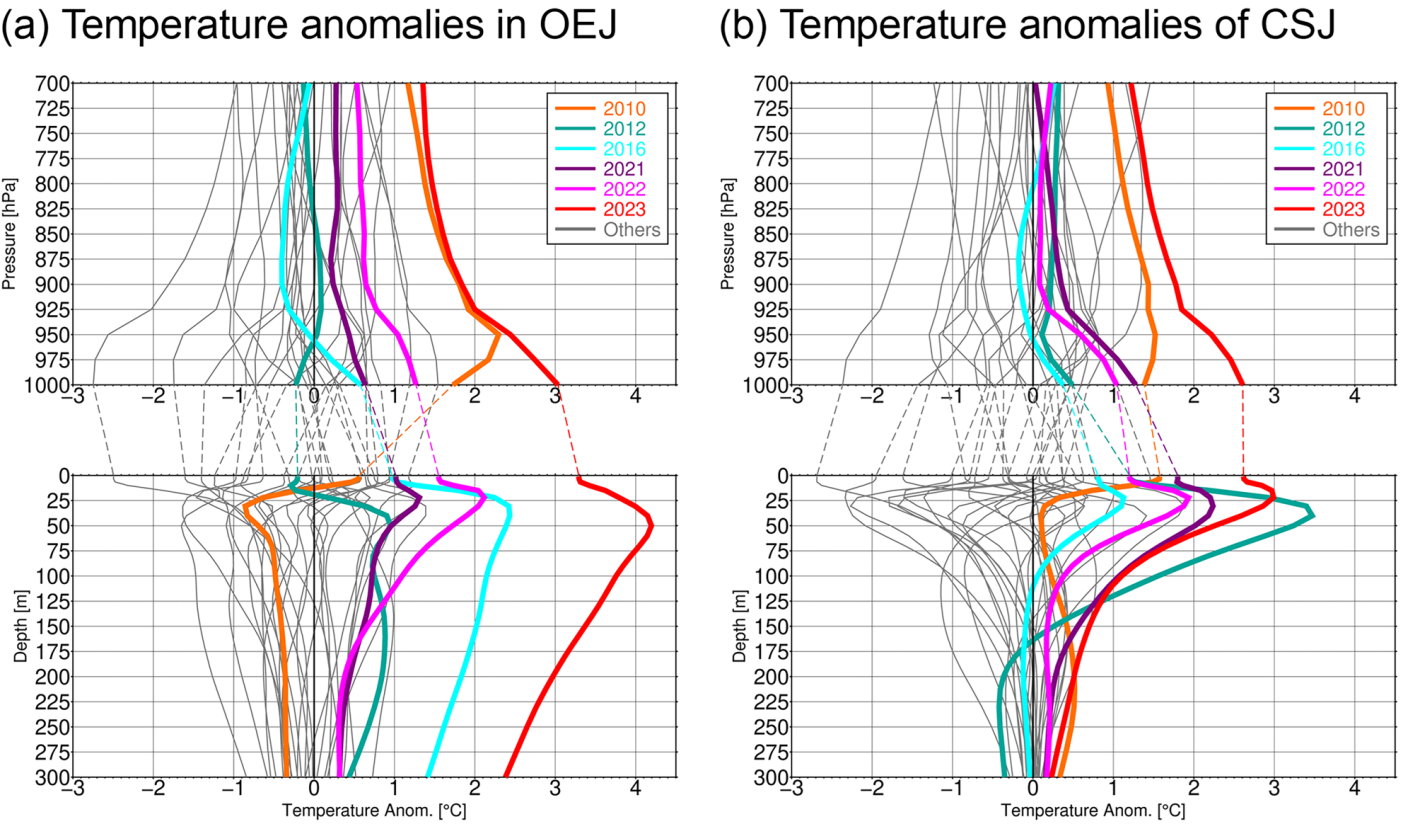
Vertical profiles of summer-mean air and ocean temperature anomalies for (**a**) OEJ and (**b**) CSJ for individual years from 1993 to 2023, where the anomalies are defined as deviations from 30-year averages for the 1993–2022 period. Years highlighted by colours are 2023 (red), 2022 (pink), 2021 (purple), 2016 (light blue) and 2010 (orange), while other years are indicated by grey lines.

In 2023 summer, the SAT anomaly averaged over Japan was the highest on record since 1898, particularly in northern Japan, where the regionally averaged SAT anomaly was also the highest on record since 1946^14^. The positive summer-mean SAT anomalies was particularly pronounced on the Pacific coast of northern Japan (Fig. 1d), suggestive of influences from the MHW. The positive SAT anomaly averaged over northern Japan persisted throughout the summer, often exceeding the corresponding 90th percentile values (Supplementary Fig. S3c). SAT and *T*_*850*_ around Japan were also well above their 30-year climatologies (Fig. 1e,f, respectively). It is striking both over OEJ and CSJ that SAT was more anomalous than *T*_*850*_. In fact, the *T*_*850*_ anomalies exceeded 2 SDs, while SAT anomalies are above 3 ~ 4 SDs, which marks a sharp contrast with another hot summer in 2010. As evident in Fig. 4, the *T*_*850*_ anomalies over the two maritime regions were comparable between 2010 and 2023, while SAT anomalies were much greater in 2023 summer than in 2010 (Fig. 2), suggestive of local influence from the MHW on the extreme summertime SAT in 2023. In fact, in both OEJ and CSJ, lower-tropospheric temperature below the 700-hPa level in 2023 was the highest since 1993, and the strongest anomalies are observed at the surface (Fig. 4), resulting in strongly reduced stratification in the atmospheric boundary layer.

As shown in Fig. 4, positive ocean temperature anomalies in 2010 were confined into the shallow surface layer above the 25-m depth, while in 2023 they extended to deeper levels. Summertime anomalies in SST and ocean temperature above the 25 m depth were also unprecedentedly high in 2023 in both OEJ and CSJ, but the whole vertical profiles of temperature anomalies differ markedly between these maritime regions. While the anomalies in CSJ were the second strongest in the 25–125 m layer and comparable to other years in the deeper layer, temperature in OEJ was unprecedentedly high since 1993 at all depths down to 300 m due to the extreme poleward meander of the KE. In fact, in-situ observations by RV Ryofu Maru of the Japan Meteorological Agency (JMA) in late July 2023 revealed that subsurface temperature anomalies around the poleward meandering KE (Fig. 1c) reached as much as 10 °C at 300–400 m depths^22^, indicating that cool Oyashio water that climatologically occupies OEJ was replaced with warmer water due to the KE meander.

Although the subsurface stratification around northern Japan is climatologically enhanced in summer due to surface warming with the mixed layer shallower than 25 m, the deep structure of the warm subsurface anomalies observed in 2023 summer suggests that the extreme SST anomalies were of the ocean dynamic origin linked to the extreme poleward KE meander. It is thus suggestive of potential impact of the MHW in 2023 on the overlying atmosphere. It is noteworthy that summer-mean water temperatures in OEJ and CSJ from the surface to around 100 m level exhibit statistically significant simultaneous correlations (with the 95% or higher confidence level) with the corresponding SAT (see Supplementary Fig. S5). The significant correlation between SAT and the subsurface temperature below the ocean mixed layer suggests that the ocean variability induced by ocean dynamics may influence the SST and SAT anomalies. The correlation coefficients in subsurface layers are less significant in CSJ than in OEJ, indicating that the influence of subsurface temperature variations onto SST and SAT tends to be weaker in CSJ than OEJ. In fact, in summer 2023, temperature anomalies at the 100 m depth and below in CSJ were rather modest in contrast to those in OEJ (Figs. 1a,b and 4). Furthermore, 100 m-depth temperature in CSJ dropped to near normal in August 2023, while positive anomalies of SST and SAT remained (Fig. 3b).

As shown in Fig. 2e, the SAT–*T*_850_ difference exhibits significant increasing tendencies during recent decades in both OEJ and CSJ. This tendency in OEJ is concurrent with an abrupt increase of summertime MHWs in the Oyashio region between 2010 and 2016^12^, which was caused by warm anticyclonic ocean eddies detached from the KE. As in 2023, in summers when subsurface temperature was significantly warmer than the climatology, static stability in the atmospheric boundary layer tends to be reduced (Fig. 4).

### Radiation and turbulent heat flux anomalies at the sea surface

**Figure 5:**
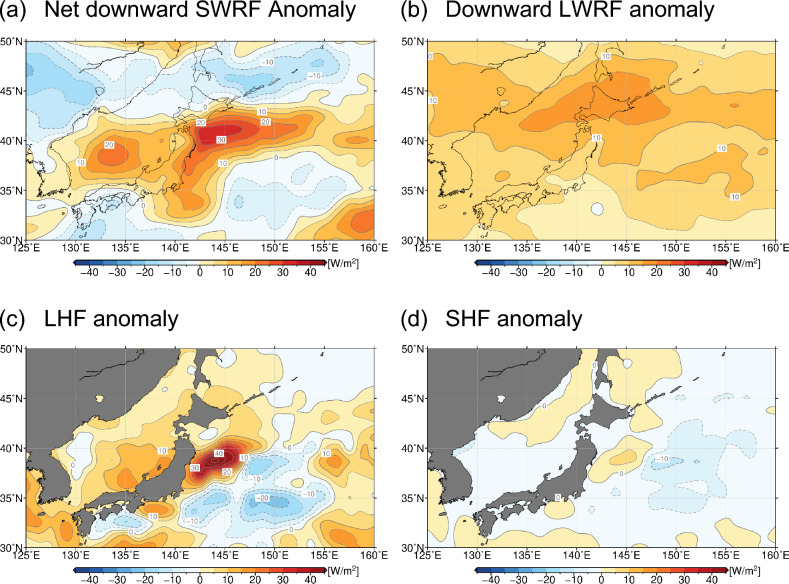
Summertime anomalies of surface radiation and turbulent heat fluxes (W/m^2^) in 2023. (**a**) Net downward shortwave (solar) radiation flux (SWRF), (**b**) downward longwave (infrared) radiation flux (LWRF), (**c**) upward latent heat flux (LHF) and (**d**) upward sensible heat flux (SHF). These maps were generated with the GMT software (ver.5.4.4; https://www.generic-mapping-tools.org/download/)^42^.

To investigate the air-sea interaction processes involved in the 2023 MHW around northern Japan, summer-mean anomalies in surface radiation and heat fluxes were evaluated (Fig. 5). Anomalies in the net downward shortwave radiation flux (SWRF; Fig. 5a) and downward longwave radiation flux (LWRF; Fig. 5b) at the surface were both positive over and around northern Japan, where anomalies in SAT and/or SST are also markedly positive, suggestive of radiative forcing onto the SAT and SST anomalies. Still, there is a possibility that the extremely warm ocean may also have exerted positive impact on SAT anomalies, which will be further examined below.

**Figure 6:**
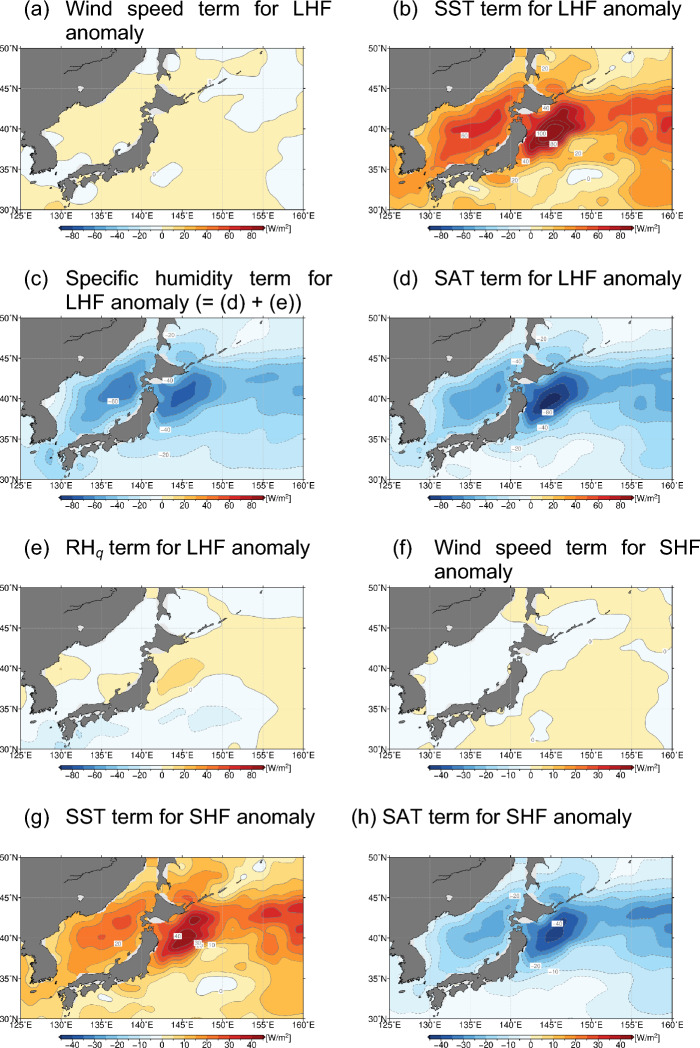
(**a**–**e**): Anomalies of surface latent heat flux (W/m^2^) in 2023 summer, due solely to (**a**) surface wind speed anomaly, (**b**) SST anomaly, (**c**) 2-m specific humidity anomaly, (**d**) SAT anomaly and (**e**) relative humidity anomaly (See the “Methods” for details). (**f**–**h**): Same as in (**a**-**e**), respectively, but for sensible heat flux anomalies (W/m^2^) due solely to (**f**) surface wind speed anomaly, (**g**) SST anomaly and (**h**) SAT anomaly. These maps were generated with the GMT software (ver.5.4.4; https://www.generic-mapping-tools.org/download/)^42^.

Latent heat flux (LHF) anomalies are also positive around northern Japan (Fig. 5c), acting to offset the enhanced SWRF and downward LWRF at the surface. The positive LHF anomalies in Fig. 5c indicate enhanced evaporation from the warm ocean, especially in OEJ, where SST was extremely high associated with the poleward meandering KE. To investigate how the warm SST anomalies influenced LHF in 2023, we performed a linear decomposition of the bulk formula of LHF (see the “Methods” section for the details) as shown in Fig. 6a–e. The LHF anomalies thus decomposed represent the contributions from local anomalies in surface wind speed (Fig. 6a), SST (Fig. 6b), SAT (Fig. 6d) and relative humidity (Fig. 6e) based on specific humidity (RH_*q*_). The most striking feature around northern Japan is the marked enhancement of evaporation by + 100 W/m^2^ due solely to the extremely high SST (Fig. 6b) sustained by the MHWs, which surpassed the counteracting contribution of − 80 W/m^2^ due to extremely high SAT (Fig. 6d). This high SAT may be attributable partly to reduced cool advection due to the diminished influence of the Okhotsk high. In addition, negative RH_*q*_ anomalies also acted to increase surface evaporation. The contribution was non-negligible in the warm OEJ region, where RH_*q*_ was reduced under the extremely high SAT even though absolute humidity at the surface was above normal. The reduced RH_*q*_ suggests less marine fog, whose formation may have been hindered due to the markedly high SST anomalies. In fact, the JMA observed the fewest fog days on record in 2023 summer at Sendai on the Pacific coast of northern Japan (see Supplementary Fig. S6a). The contribution from anomalous surface wind speed was found smaller than other contributions. Still, surface winds slightly intensified around northern Japan acted to enhance evaporation, which is consistent with more active turbulent mixing of wind momentum under the reduced near-surface static stability and can be regarded as an indirect impact of the warm SST anomalies.

Though smaller in magnitude than the LHF anomalies, sensible heat flux (SHF) anomalies in summer 2023 were also positive in OEJ (Fig. 5d), where SHF is climatologically weak in summer. This indicates warming of surface air directly by the underlying ocean in the presence of warm SST anomalies, resulting in the reduced static stability in the atmospheric boundary layer in 2023 summer (Fig. 4). A similar linear decomposition reveals that the positive SHF anomalies in OEJ were accounted for mainly by the unprecedentedly high SST (Fig. 6g), surpassing the offsetting contribution by the extremely high SAT (Fig. 6h). Reflecting the stronger SST anomaly than the SAT anomaly (Fig. 4a), this positive SHF anomaly is suggestive of thermodynamic forcing by the MHW. The contribution of surface wind speed anomalies was found marginal (Fig. 6f).

### Influence of warm SST anomalies on reduced low-level cloud cover

**Figure 7:**
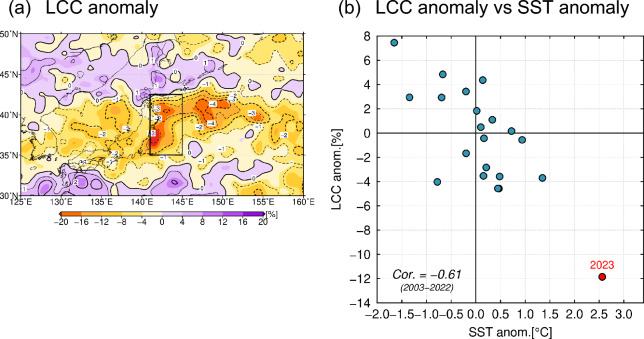
(**a**) Summer-mean anomalies (shaded) and normalized anomalies (contoured) by its standard deviation of interannual variations of low-level cloud cover (LCC, %), in 2023. Thick and thin contour intervals are 2 and 1, respectively. The map was generated with the GMT software (ver.5.4.4; https://www.generic-mapping-tools.org/download/)^42^. (**b**) Scatter plot of summer-mean anomalies in SST (°C) versus LCC (%), both averaged within the domain off the east coast of Japan (35°–42.5°N, 141°–145°E, denoted with the black rectangular in (**a**)). Red indicates 2023 summer. The corresponding correlation coefficient for the 2003–2022 period is shown at the lower left of the panel. The anomalies are defined as deviations from 30-year averages with the 2003–2022 period.

The positive anomaly of summer-mean downward SWRF around northern Japan in 2023 (Fig. 5a) can be due to anomalous low-level cloud cover (LCC). Climatologically over the summertime mid-latitude western North Pacific, low-level clouds are known to exert net radiative cooling due to their high albedo in excess of their greenhouse effect^23,24^. Over that region, low-level stratiform cloud decks predominate under the climatologically high near-surface static stability due to the warm monsoonal southerlies over the cool Oyashio water on the poleward side of the oceanic frontal zone (see Supplementary Fig. S7 for the summer-mean LCC distributions in 2023 and for climatology). We have confirmed significant negative correlation (at the 95% confidence level) between LCC and SST off the east coast of Japan (Fig. 7b), where strong SST anomalies generated by the oceanic front variability^25^ can modify the near-surface static stability.

In 2023, marine LCC was substantially less than its climatology around northern Japan (Fig. 7a), where the underlying SSTs were extremely above-normal (Fig. 1a), particularly off the east coast of Japan. The reduced LCC is consistent with the fewest fog days observed at Sendai (Supplementary Fig. S6a). Both the positive SST and negative LCC anomalies off the east coast of Japan in 2023 were exceptional since the beginning of the MODIS cloud data in 2003 (Fig. 7b). The negative LCC anomaly in summer 2023 (Fig. 7a) collocated with the warm SST anomaly (Fig. 1a) that had persisted since the preceding April associated with the poleward meandering KE (Fig. 3a) is suggestive of potential impact of the latter on the former, possibly through reduced lower-tropospheric stratification (Fig. 4a). This process constitutes a positive cloud radiative feedback on SST^5,6,24,26^, i.e., the reduced LCC enhanced surface insolation to sustain the warm SST anomaly, which in turn acted to reduce LCC by lowering lower-tropospheric stratification. The warm SST anomaly around northern Japan maintained partially through the LCC-SST feedback contributed in part to enhanced surface evaporation (Fig. 5c), which may have been involved in another possible feedback as discussed below.

### Influence of enhanced evaporation and moisture

**Figure 8:**
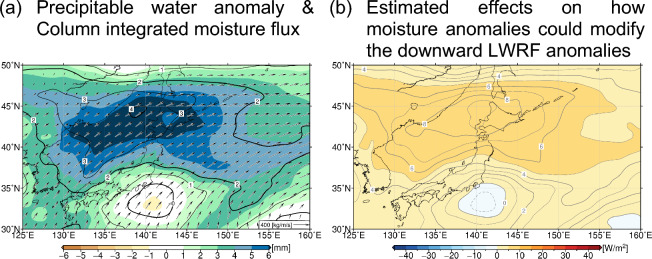
(**a**) Summer-mean anomalies (shaded) and normalized anomalies (contoured) by its standard deviation of interannual variations of column integrated water vapour (precipitable water, mm) in 2023. Thick and thin contour intervals are 2 and 1, respectively. The summer-mean moisture flux (kg/m/s) is indicated with arrows. (**b**) Estimated effects on how moisture anomalies could modify the downward LWRF anomalies (W/m^2^) (See the “Methods” for detail). The contour intervals are 1 W/m^2^. These maps were generated with the GMT software (ver.5.4.4; https://www.generic-mapping-tools.org/download/)^42^.

One of the prominent features in the summer of 2023 is substantially increased precipitable water (i.e., column-integrated water vapour) over and around northern Japan. In fact, precipitable water anomaly exceeded 3 SDs in CSJ and to its north (Fig. 8a). The spatial distribution of the precipitable water anomalies nearly coincided with that of the surface downward LWRF anomaly (Fig. 5b), implying that abundant moisture locally enhanced the greenhouse effect. To examine this, we adopted a diagnosis method^27^ (see the “Methods” section for details) as in the previous studies^15,16^ that argued the impact by the Kuroshio large meander on the summertime hot and humid conditions in adjacent regions. Our simple estimate indicates that an approximately 6 ~ 9 W/m^2^ increase of surface downward LWRF around northern Japan under the cloud-free condition, including over the landmass (Fig. 8b), accounting for more than half of the analysed values (Fig. 5b). This implies that the exceptionally humid condition may have exerted substantial impact on the SAT anomalies via enhanced greenhouse effect.

The abundant moisture in 2023 summer was transported largely from the subtropics (Fig. 8a), but there may be an additional contribution from the enhanced surface evaporation around northern Japan (Fig. 5c), where large specific humidity anomalies were confined in the near-surface layer (not shown) in corresponding to the air temperature anomaly profiles (Fig. 4). Along the west coast of northern Japan, moistening tendency during 2023 summer was overall collocated with enhanced evaporation and the anomalous divergence of the column-integrated moisture flux (see Supplementary Fig. S8). Although the reduced LCC (Fig. 7a) acted to decrease the downward LWRF, the positive LWRF anomaly shown in Fig. 5b is indicative of the surpassed contribution from the enhanced moisture, especially in the extremely warm near-surface layer, which acted to maintain the markedly warm SST underneath through positive feedback.

## Conclusion and discussion

This study is among the first to analyse atmospheric and oceanic conditions during the unprecedented heatwave over Japan in summer 2023 and thereby reveal potential impact of the pronounced MHW event with extremely warm SST around northern Japan. We have found that the MHW east of Japan was associated with the marked poleward meander of the KE, which had lasted since the preceding spring, and thus subsurface ocean temperature was also unprecedentedly high down to at least 300 m depth. This deep structure of the summertime ocean temperature anomalies indicates that cool Oyashio water that climatologically occupies this region was replaced with much warmer water due to the markedly meandering KE that had persisted since late spring. Although the whole lower troposphere was unprecedentedly warm, the temperature anomalies were particularly enhanced below the 925-hPa level and collocated with the record-setting positive anomalies in SST and subsurface ocean temperature. The deep subsurface anomalies and the reduced stratification in the atmospheric boundary layer both suggest potential impact of the MHW in association with the large KE meander generated mainly through ocean dynamics on the atmospheric heatwave in 2023.

Our analysis of surface radiation and turbulent heat flux anomalies indicates thermal forcing onto SAT by enhanced insolation and downward long-wave radiation at the surface, but there could also be local oceanic effects behind these radiation flux anomalies. We found a prominent increase of evaporation from the ocean around northern Japan, especially in the northward bulge of the KE, through a primary contribution from the extreme warm SST anomaly and a secondary contribution of reduced relative humidity. We also found upward sensible heat flux anomalies around the northward meandering KE, indicative of direct warming of the atmospheric boundary layer by the extremely warm ocean and the resultant reduction of near-surface stratification.

Behind the enhanced insolation there could be a local oceanic effect through cloud-SST feedback. The reduced lower-tropospheric stratification over the warm SST anomalies was unfavourable for low-cloud formation and thus leading to the increased insolation, which in turn acted to reinforce the warm SST anomalies. In fact, satellite-measured low-cloud cover was unprecedentedly low in 2023 summer off the east coast of Japan, where SST was also record high, which suggests the positive cloud-SST feedback operative for the maintenance of those anomalies.

In addition, positive feedback through increased moisture over the warm SST anomalies could be behind the intensified surface downward long-wave radiation. Its distribution was found to correspond well with that of positive precipitable water anomalies, which could account for more than 50% of the increase in downward long-wave radiation under the cloud-free condition. In addition to the moisture transport from the subtropics, the enhanced evaporation from the warmed sea surface around northern Japan also contributed to the moistening of the atmosphere especially along the west coast of northern Japan. This is suggestive of the increased greenhouse effect in 2023 summer by the humidification of the extremely warm lower troposphere due partly to the enhanced moisture supply from the warmed ocean nearby. The anomalous greenhouse effect surpassed the counteracting effect of the reduced low-cloud cover to yield the net increase of downward long-wave radiation, acting to reinforce the warm SST anomalies.

The warm SST anomaly in 2023 summer could influence SAT over the northern Japan landmass to some extent not only through enhanced greenhouse effect by moistening the lower troposphere but also through anomalous near-surface warm advection (see Supplementary Fig. S9). This can approximately be decomposed into to a wind anomaly term and a temperature anomaly term as.

$$\left(-{{\varvec{u}}}_{\text{H}}{\nabla }_{\text{H}}T\right)^{\prime} \sim -{{{\varvec{u}}}^{\prime}}_{\text{H}}{\nabla }_{\text{H}}\overline{T }- {\overline{{\varvec{u}}} }_{\text{H}}{\nabla }_{\text{H}}T^{\prime}$$

where a prime signifies an anomaly of a given variable, an overbar its climatological mean, ${\varvec{u}}$_H_ horizontal wind vector, and $T$ temperature, respectively. At the 975-hPa level, the net anomalous warm advection around northern Japan was attributable primarily to the anomalous southerlies between 35° and 45°N (Supplementary Fig. S9a), while the contribution from temperature anomalies was non-negligible around 40 ~ 45°N. This is suggestive of downstream influence of the warm SAT anomalies over the MHW regions. Furthermore, though not necessarily obvious in the seasonal-mean field, cool advection by sea breeze seemed to be reduced under the pronounced warm SST anomalies. Sea breeze is known to peak generally in the afternoon when land-sea thermal contrast tends to maximize in the diurnal cycle. As an example, we examined summer-mean easterly sea breeze at 0600 UTC (1500 local time) on the east coast of Japan adjacent to the extremely warm OEJ. Supplementary Fig. S10 shows the relationship of the easterly or land-sea zonal temperature gradient with the temperature advection, based on conditional sampling only when the surface zonal wind component was easterly. Apparently, the cool advection in summer is positively correlated with both the easterly wind speed and the thermal contrast. In 2023, cool advection was weaker than its climatological mean under the weakened easterly sea breeze and reduced land-sea thermal contrast due to the extremely high SST.

The present study is one of the first attempts to clarify how the extremely warm ocean due to the large KE meander locally influenced the record atmospheric heatwave around northern Japan in summer 2023. Our study will promote future observational and numerical studies to elucidate the specific processes involved in the interactions between the unprecedented marine and atmospheric heatwaves in 2023 summer around Japan, which will be informative to future studies in other heatwave events in other maritime regions over the globe. Specifically, our findings can also provide valuable insights into air-sea interactions around summertime mid-latitude oceanic frontal zones along the western boundary currents, as we emphasized cloud-SST feedback via anomalous insolation and sensible heating as well as water vapour feedback through anomalous evaporation and greenhouse effect.

As the global warming progresses, the intensity and frequency of MHWs will continue to increase^3^. Taking into account the unique and prominent features inherent to the vicinity of Japan and other western boundary current regions that the warming trend of SST is much greater than that of the global mean^28^, it will be of particular importance to understand and predict what local impacts of MHWs can influence atmospheric heatwaves around those regions, as well as the mechanisms of triggering, maintaining, and amplifying the MHWs themselves. In this paper we evaluated the contribution of anomalous air-sea heat exchange to the ocean mixed-layer temperature, but further work is required for direct assessment of the contribution of oceanic dynamical process, such as advection and vertical mixing. For instance, intensified surface wind speed and reduced stratification around the mixed-layer bottom (see Supplementary Fig. S11) were likely favourable for enhanced vertical mixing and entrainment to moderate mixed-layer warming off northern Japan. The processes discussed in this paper need to be validated further with quantitative analyses, for example, through numerical model experiments. Better sub-seasonal to seasonal prediction based on deeper understanding of such mid-latitude local air-sea interactions could contribute anticipatory actions for future extreme high temperature events.

## Methods

### Data

The main data for investigating atmospheric circulation field in this study is the monthly and daily data of the Japanese Reanalysis Three Quarters of a Century (JRA-3Q^29^) with horizontal resolution of 1.25° × 1.25°. SST analysis data are taken from the Merged Satellite and In-situ Data Global Daily Sea Surface Temperature (MGDSST^30^) on a grid of 0.25° × 0.25°. We use analysed data of JRA-3Q and MGDSST data after June 1985, when the latter was used as the lower boundary conditions for the JRA-3Q calculation. The version 2 of Centennial In Situ Observation-Based Estimates of the Variability of SST and Marine Meteorological Variables (COBE-SST2^31^) data set is also used for the analysis of long-term historical SST since the early twentieth century. Oceanic subsurface analysis from 1993 onwards data have been obtained from the JMA Multivariate Ocean Variational Estimation/Meteorological Research Institute Community Ocean Model for the North Pacific (MOVE/MRI.COM-NPR^32,33^; hereafter MOVE-NPR). Its horizontal resolution is variable and ~ 10 km around Japan.

The collection 6.2 level-3 daily cloud product (MCD06COSP_D3_MODIS^34^), which combines the Moderate Resolution Imaging Spectroradiometer (MODIS) on *Terra* and *Aqua*, is used to estimate LCC, where clouds with top pressures higher than 680 hPa are defined as low-level clouds. The spatial resolution is 1° × 1°. We used the data from 2003 onwards. We derive temporal averages of LCC as pixel-count weighted averages for each grid. Owing to difficulties in detecting low-level clouds with passive sensors from space due to their overlaps with mid- and/or high-level clouds, we calculated LCC assuming random overlap^35^. Weather station data of SAT and fog days observed by the JMA are also used.

Daily and monthly climatologies are defined as averages for the period from 1991 to 2020 except for MODIS data and MOVE-NPR. The climatology for MODIS data is defined as averages from 2003 to 2022 and MOVE-NPR data is from 1993 to 2022. Anomalies of a given variable are defined locally as deviations from its climatology.

### Marine heatwave

Following Hobday et al*.*^1^, we define a MHW as an event in which area-averaged SST stays above the 90th percentile values for at least five consecutive days with a gap, if any, of two days or less between the subsequent warm days. The 90th percentile threshold is based on 11-day running mean SST from 1991 to 2020, and then smoothed by 31-day running mean. An intensity-dependent classification of MHWs proposed by Hobday et al*.*^21^ is used in this study. Specifically, SST anomalies that exceed a unit, twice, three times and four times of the 90th percentile anomaly from the climatological normal are rated Categories I (modest), II (strong), III (severe) and IV (extreme) events, respectively.

### Temperature tendency in the ocean mixed layer by air-sea heat exchange

Contributions from air-sea heat exchange to the ocean mixed layer temperature are estimated as in previous studies (e.g., Oliver et al*.*^9^; Noh et al*.*^38^) but under a crude assumption of the fixed ocean mixed layer depth. The temperature tendency due to air-sea heat fluxes can be expressed as follows:

$$\frac{\text{SWRF}+\text{LWRF}+\text{LHF}+\text{SHF}-{\text{SWRF}}_{-h}}{{C}_{\text{p}}\rho h},$$

where the downward fluxes are defined positive, and mean density $\rho$ and specific heat capacity ${C}_{\text{p}}$ of sea water are prescribed as 1025 kg/m^3^ and 3940 J/(kg K), respectively. The ocean mixed layer depth $h$ is fixed to 10 m. The attenuated shortwave radiation at the mixed layer bottom ${\text{SWRF}}_{-h}$ is estimated as follows^39,40^:

$${\text{SWRF}}_{-h}=\text{SWRF}\left[{R\text{e}}^{-h/{\gamma }_{1}}+\left(1-R\right){\text{e}}^{-h/{\gamma }_{2}}\right],$$

with attenuation parameters $R=0.77$, ${\gamma }_{1}=1.5$ m and ${\gamma }_{2}=14$ m. They correspond to Jerlov water Type II^41^ as in Qiu and Kelly^40^, who conducted a heat budget analysis in the KE region.

### Linear decomposition of sensible and latent heat flux anomalies

LHF and SHF anomalies can be linearly decomposed into several contributing terms based on the Bulk formulae:

$$\text{LHF}={\rho }_{a}L{C}_{E}{W}_{10}\left({q}_{\text{sat}}\left({T}_{s}\right)-{q}_{a}\right) \, \text{and}$$

$$\text{SHF}={\rho }_{a}{C}_{p}{C}_{H}{W}_{10}({T}_{s}-{T}_{a})$$

where ${W}_{10}$*, *${T}_{s}$*,* ${T}_{a}$ are 10-m scalar wind speed, SST, SAT, respectively. ${q}_{\text{sat}}\left({T}_{s}\right)$ is saturation specific humidity at ${T}_{s}$. ${q}_{a}$ is 2-m specific humidity. ${\rho }_{a}$, $L$ and ${C}_{p}$, are the density of air, latent heat of vaporisation and the specific heat of air at constant pressure, respectively. We let the bulk transfer coefficients ${C}_{E}$ and ${C}_{H}$ be $1.3\times {10}^{-3}$^36^. LHF and SHF anomalies were linearly decomposed as below, following Tanimoto et al.^37^ but in a slightly modified manner:

$$\Delta \text{LHF} \sim \overline{{\rho }_{a}}L{C}_{E}{[\Delta W}_{10}\left({q}_{\text{sat}}\left(\overline{{T}_{s}}\right)-\overline{{q}_{a}}\right)+ \overline{{W}_{10}}\Delta {{q}}_{\text{sat}}\left({T}_{s}\right)- \overline{{W}_{10}}\Delta {q}_{\text{a}}]$$

$$\sim \overline{{\rho }_{a}}L{C}_{E}{[\Delta W}_{10}\left({q}_{\text{sat}}\left(\overline{{T}_{s}}\right)-\overline{{q}_{a}}\right)+ \overline{{W}_{10}}\Delta {q}_{\text{sat}}\left({T}_{s}\right)-\overline{{W}_{10}}\Delta {q}_{\text{sat}}\left({T}_{a}\right)\overline{{\text{RH}}_{q}}-\overline{{W}_{10}}{q}_{\text{sat}}\left(\overline{{T}_{a}}\right)\Delta {\text{RH}}_{q}]$$

$$\Delta \text{SHF }\sim \overline{{\rho }_{a}}{C}_{p}{C}_{H}{[\Delta W}_{10}\left(\overline{{T}_{s}}-\overline{{T}_{a}}\right)+ \overline{{W}_{10}}\Delta {T}_{s}- \overline{{W}_{10}}\Delta {T}_{a}]$$

where ${\text{RH}}_{q}\equiv {q}_{a}/{q}_{\text{sat}}\left({T}_{a}\right)$ is relative humidity based on the specific humidity but not the water vapour pressure. Overbars and $\Delta$ denote the climatologies and anomalies, respectively.

### Influences of precipitable water on the downward LWR

As similar to the previous studies^15,16^, we estimated the influences of precipitable water on the surface downward longwave radiation (LWRF) for the clear sky^27^ by the following formula:

$$\text{LWRF for clear sky}=\left\{1-\left(1+u\right)\text{exp}\left[-{\left(1.2+3u\right)}^\frac{1}{2}\right]\right\}\sigma {T}_{a}^{4}\equiv f(u)\sigma {T}_{a}^{4}$$

where $u=0.1\times \text{precipitable water }[\text{kg}/{\text{m}}^{2}]$ and $\sigma$ is the Stefan–Boltzmann constant. As the term inside of the curly bracket is dependent only on precipitable water, we denote it as $f(u)$ for simplicity. To examine how surface LWRF changes associated with precipitable water anomalies, we calculated $\Delta f\sigma {\overline{{T}_{a}}}^{4}$. Please note that this estimation is valid only under the cloud-free condition. In reality, there would be other contributing factors including radiation from clouds at various altitudes.

### Supplementary Information

Supplementary Figures.

## Data Availability

The monthly and daily JRA-3Q reanalysis data can be downloaded from the Data Integration and System (DIAS), which was developed and operated by a project supported by the Japan’s Ministry of Education, Culture, Sports, Science and Technology (10.20783/DIAS.645). The MGDSST and COBE-SST2 dataset are available from the North-East Asian Regional—GOOS (the Global Ocean Observing System) website (https://www.data.jma.go.jp/goos/data/database.html). The MOVE-NPR dataset can be available from the corresponding author on reasonable request. The MCD06COSP_D3_MODIS data is available from NASA (10.5067/MODIS/MCD06COSP_D3_MODIS.062). Weather station data of SAT and foggy days in Japan are available from the JMA website (https://www.data.jma.go.jp/stats/etrn/index.php, in Japanese).
