# Supplementary material for: Impact of an unprecedented marine heatwave on extremely hot summer over Northern Japan in 2023
Source: Sci Rep. 2024 Jul 19;14:16100. doi: 10.1038/s41598-024-65291-y (PMC11271639; doi:10.1038/s41598-024-65291-y)
Supplement: Supplementary file 1 — Supplementary Figures. [file 41598_2024_65291_MOESM1_ESM.pdf]

Supplementary Figures

Impact of an Unprecedented Marine Heatwave on Extremely Hot Summer over Northern Japan in 2023

Hirotaka Sato, Kazuto Takemura, Akira Ito, Takafumi Umeda, Shuhei Maeda, Youichi Tanimoto, Masami Nonaka, and Hisashi Nakamura

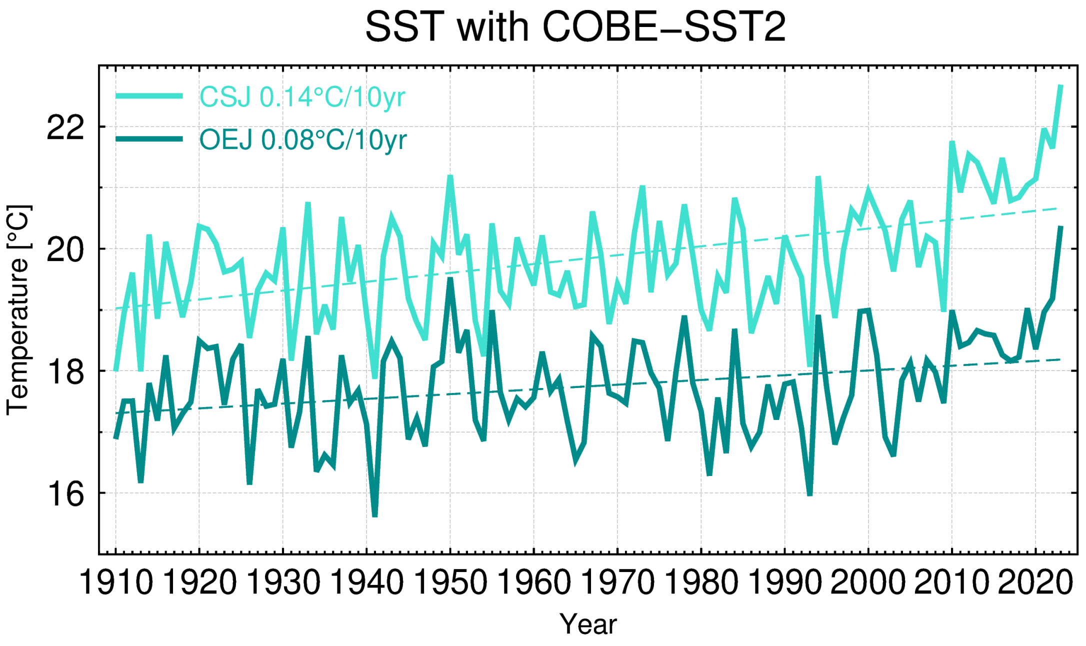

Supplementary Figure S1. Timeseries of summer-mean SST averaged in Oyashio region east of northern Japan (OEJ; 37.5°–42.5°N, 142°–149°E, green rectangular in Fig. 1) and central portion of the Sea of Japan (CSJ; 37.5°–42.5°N, 133°–140°E, light blue rectangular in Fig. 1) with COBE-SST2 dataset. Dashed lines indicate linear trends, shown if statistically significant at the 95% confidence level based on Student's *t*-test. The corresponding linear trend values of each area are shown at the top left of the panel. Note that values in the 1940s are less reliable due to lack of observational data.

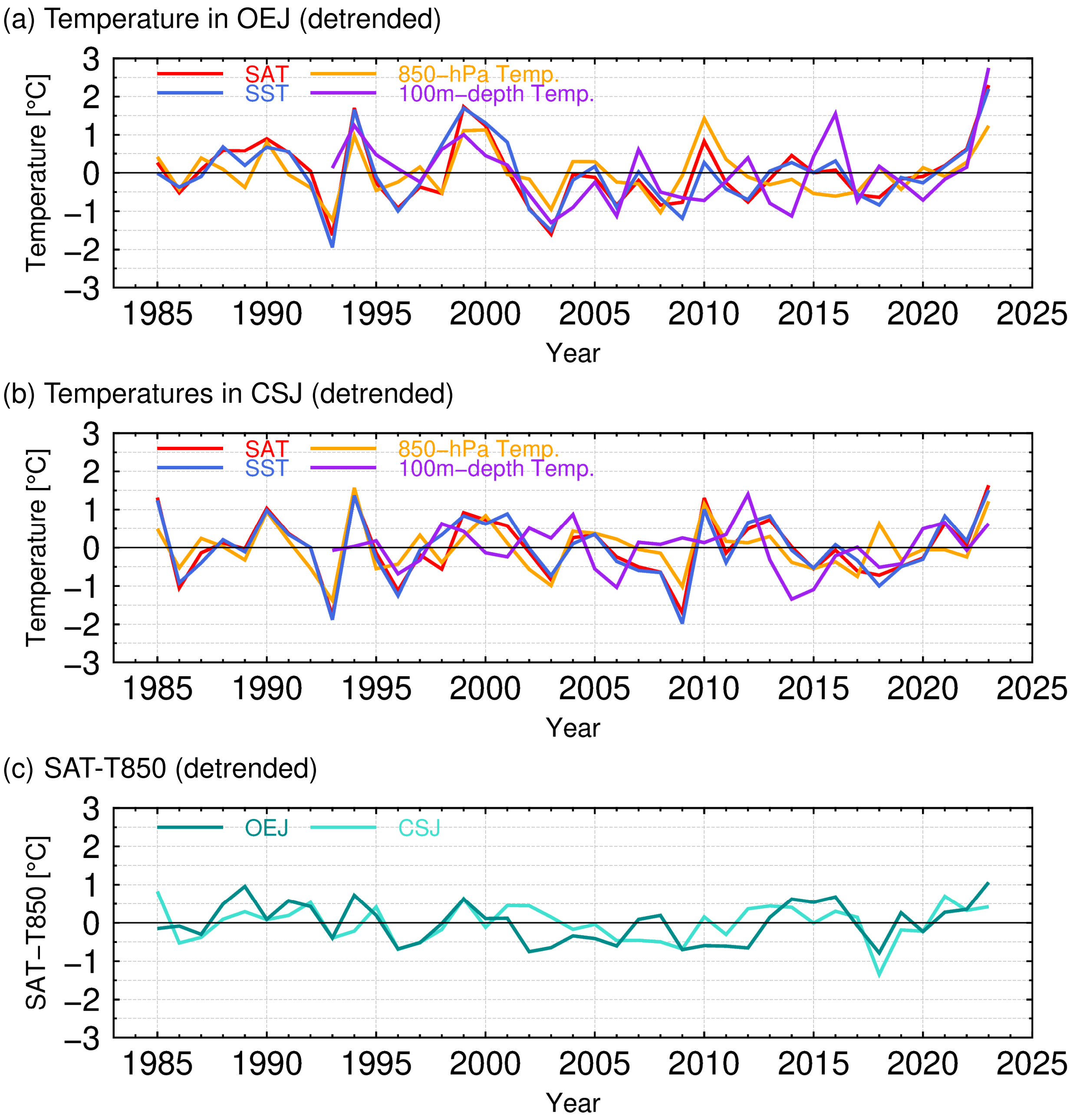

Supplementary Figure S2. Same as in Fig. 2, but for detrended data. Timeseries of summer-mean SAT (red), SST (blue), 850-hPa temperature (orange) and 100m-depth temperature (purple) averaged within (a) Oyashio region east of northern Japan (OEJ; 37.5°–42.5°N, 142°–149°E, green rectangular in Fig. 1) and (b) central portion of the Sea of Japan (CSJ; 37.5°–42.5°N, 133°–140°E, light blue rectangular in Fig. 1). (c) Timeseries of summer-mean SAT minus 850-hPa temperature for OEJ (green) and CSJ (light blue).

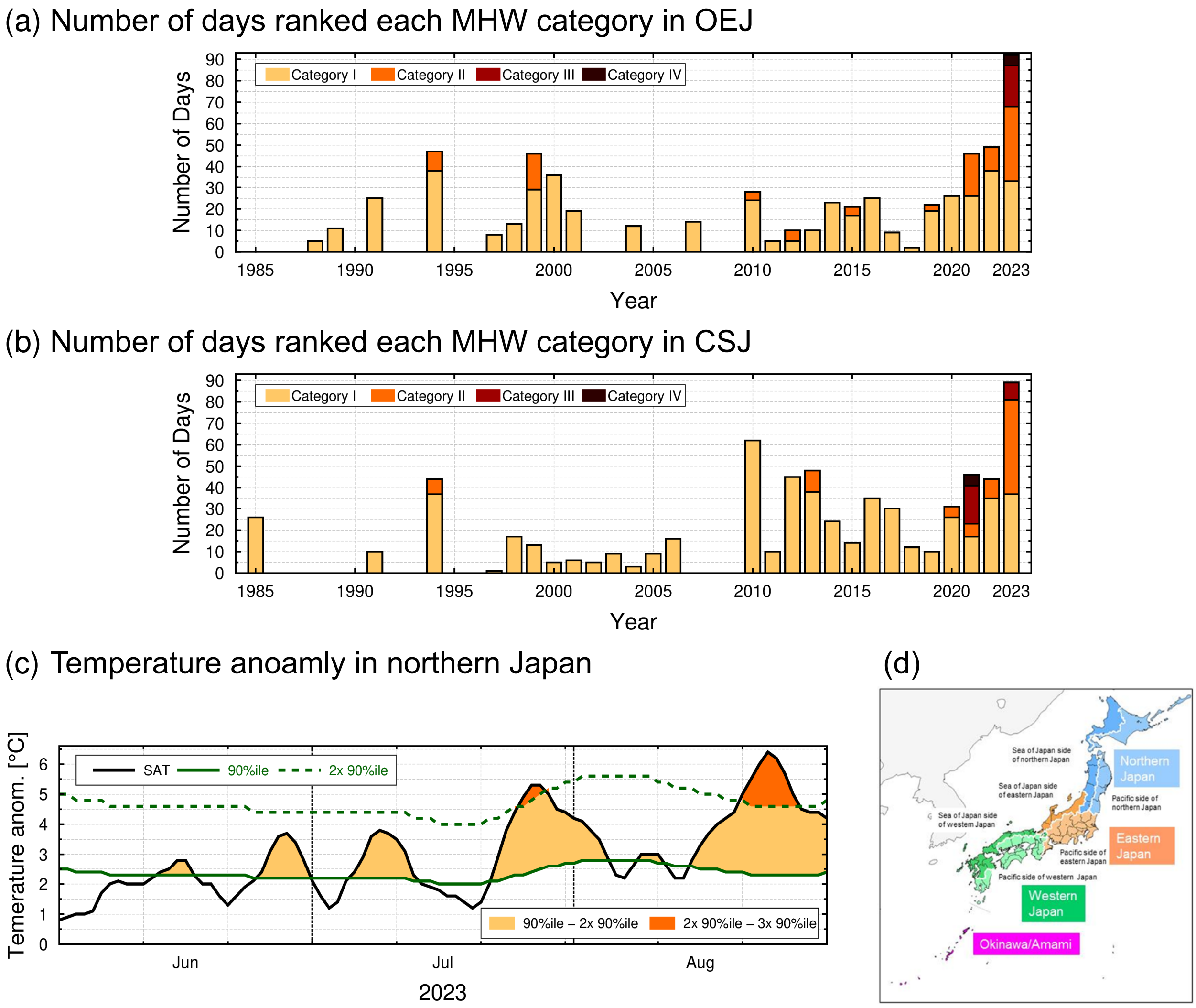

Supplementary Figure S3. Metrics of marine heatwave (MHW) in OEJ (a) and CSJ (b). (a-b) Number of days of the individual MHW categories I (modest), II (strong), III (severe) and IV (extreme) in each summer (June-July-August) from 1985. (c) Timeseries of 5-day running mean SAT anomalies (°C; black line) averaged over northern Japan, based on station observations. The 90th percentile of the SAT (°C; green solid line; 90%ile) calculated for the 30-year period of 1991-2020 and twice the 90th percentile difference from the climatological normal (°C, green dashed line: 2x 90%ile) are also shown with the corresponding categories for individual days indicated with colours. (d) Map of Japan. Blue-coloured region corresponds to northern Japan. The map is from *Climate Change Monitoring Report 2022* (https://www.jma.go.jp/jma/en/NMHS/ccmr/ccmr2022.pdf) by the Japan Meteorological Agency.

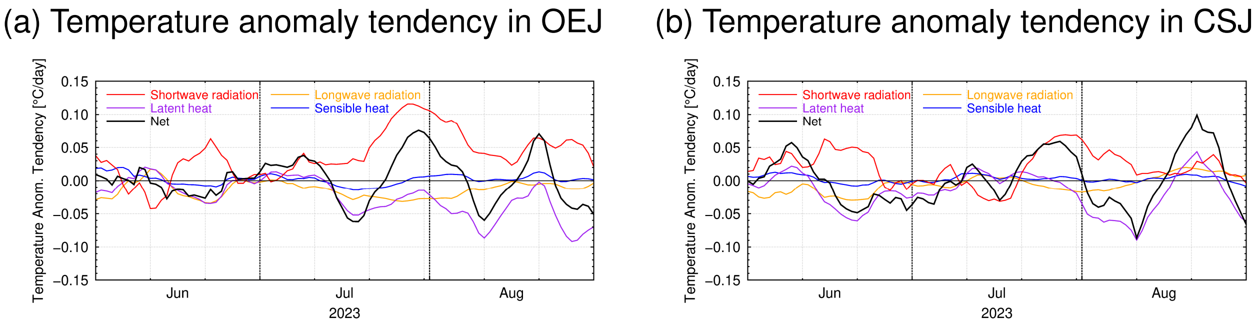

Supplementary Figure S4. Tendency of 11-day running mean temperature anomaly in the ocean mixed layer (°C/day) due solely to each of the air-sea heat flux anomalies in (a) OEJ and (b) CSJ. Red, orange, purple, blue and black lines indicate contributions of shortwave radiation, longwave radiation, latent and sensible heat fluxes as well as net air-sea exchange, respectively. The anomalies are all defined as deviations from 30-year averages for the 1993–2022 period.

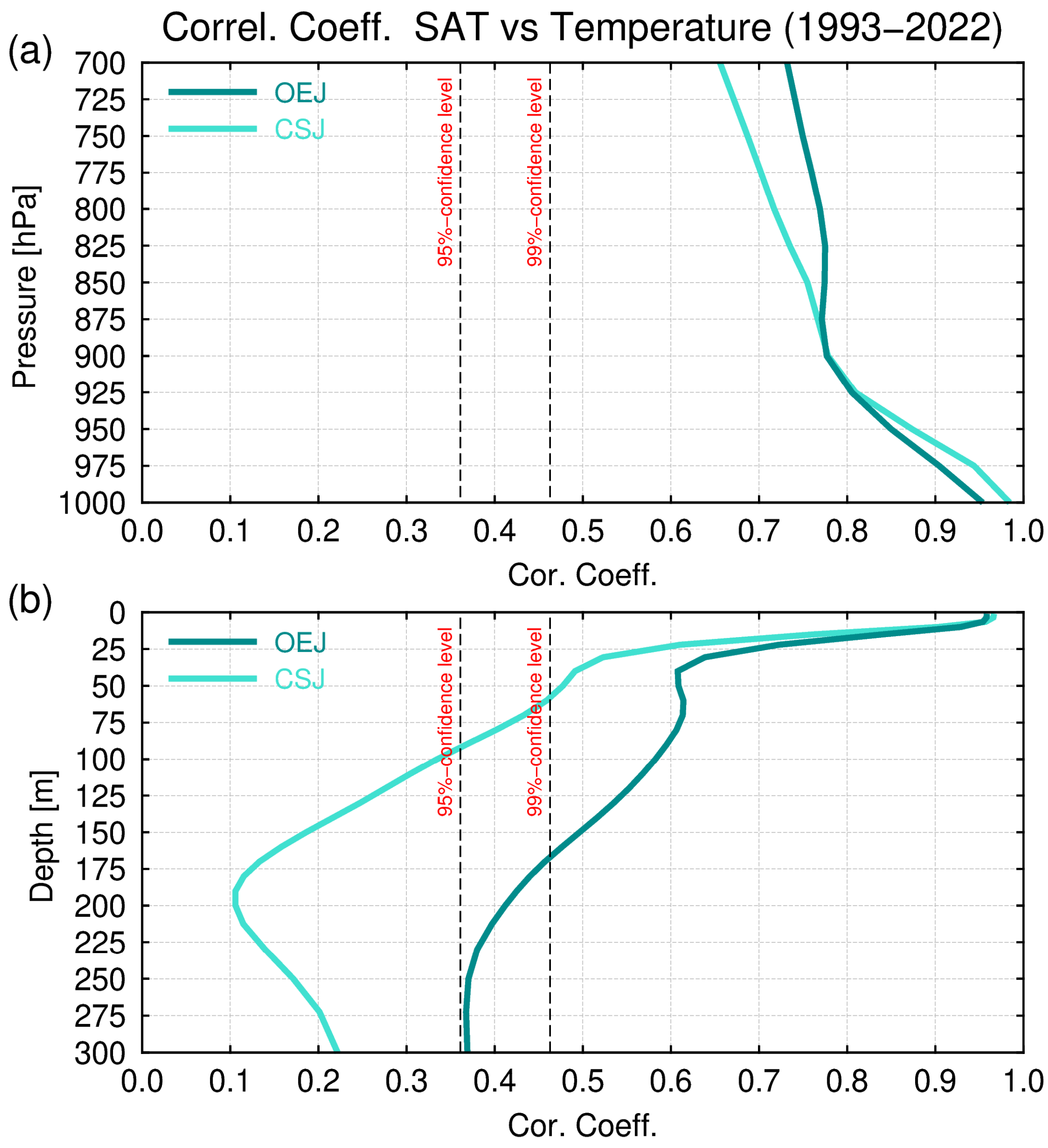

Supplementary Figure S5. Vertical profiles of the correlation coefficients between summer-mean SAT and temperatures in (a) the lower troposphere and (b) the ocean sub-surface layers for the OEJ (green) and CSJ (light blue) regions, for the period 1993–2022. The 95% and 99% confidence levels based on Student's *t*-test are indicated with dashed lines.

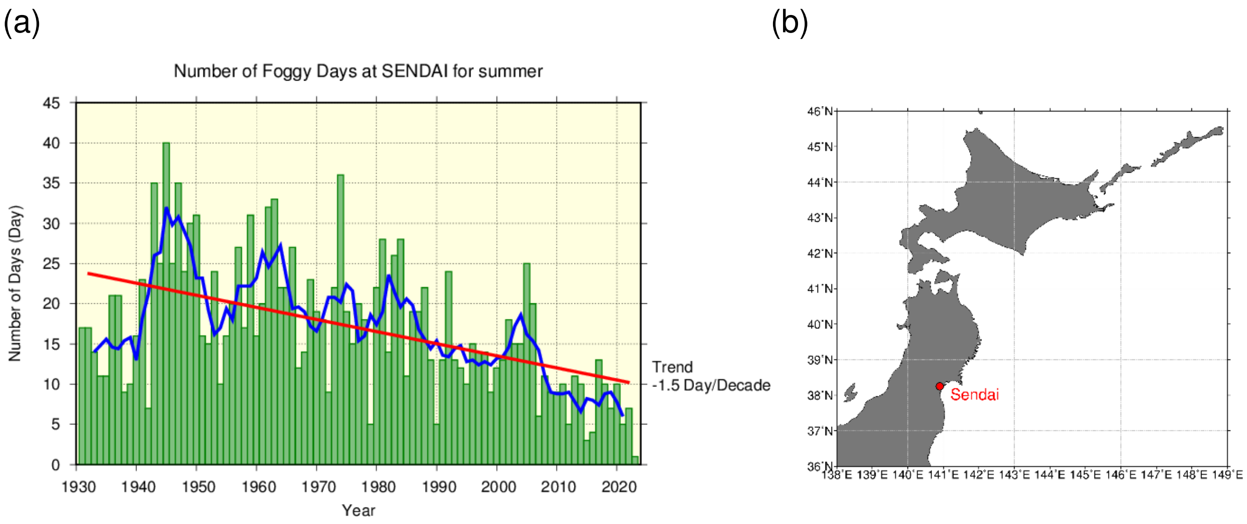

Supplementary Figure S6. (a) Interannual timeseries of the number of fog days observed at Sendai during summer (green bars). Blue and red lines denote the five-year running mean and the linear trend, respectively. (b) Map of northern Japan. The red dot indicates the location of Sendai. The map was generated with the Generic Mapping Tools software (ver.5.4.4; https://www.generic-mapping-tools.org/download/).

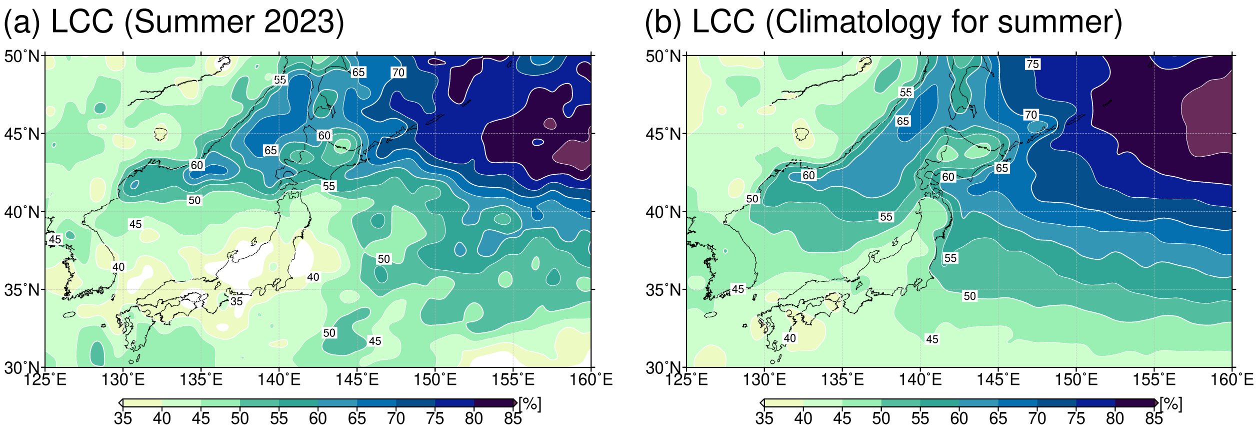

Supplementary Figure S7. Horizontal summer-mean distributions of (a) LCC (%) in 2023 and (b) the climatology. These maps were generated with the GMT software (ver.5.4.4; https://www.generic-mapping-tools.org/download/).

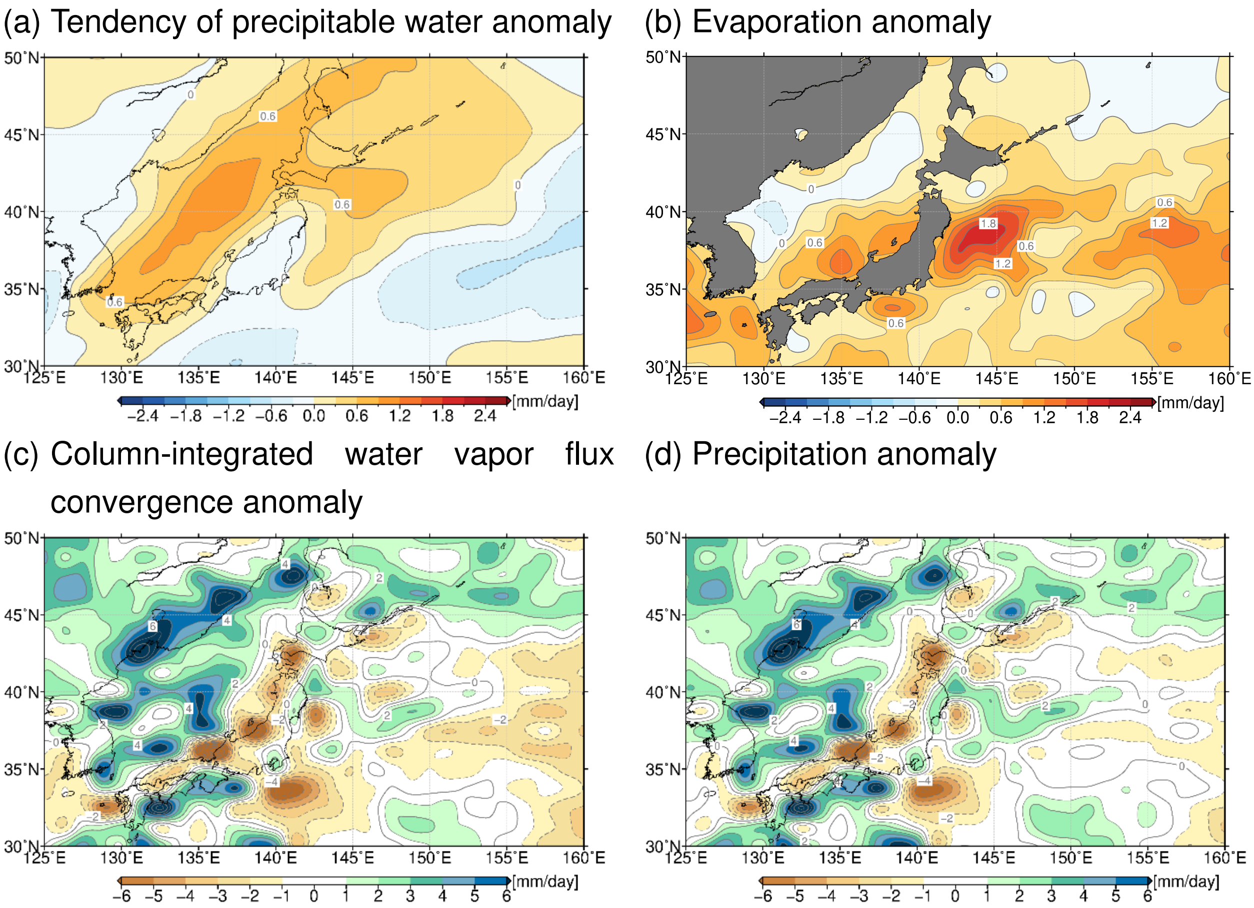

Supplementary Figure S8. (a) Tendency of anomalous precipitable water (mm/day), (b) anomalous evaporation (mm/day), (c) anomalous convergence of column-integrated moisture flux (mm/day) and (d) anomalous precipitation (mm/day) (calculated as the residuals of moisture budget in (a)–(c)) in the period from 21 July 2023 to 31 August 2023. These maps were generated with the GMT software (ver.5.4.4; https://www.generic-mapping-tools.org/download/).

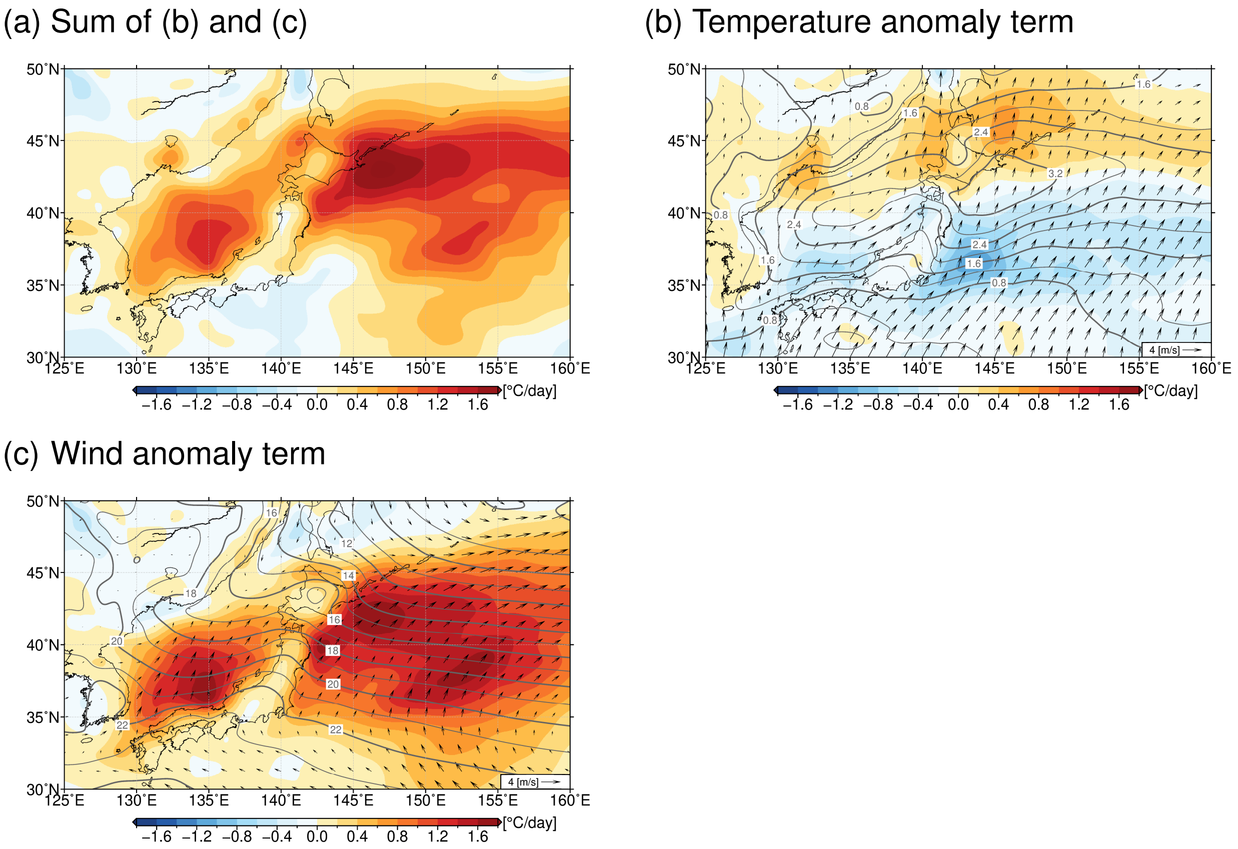

Supplementary Figure S9. (a) Anomalies of 975-hPa temperature advection (°C/day) averaged in 2023 summer. (b, c) Same as in (a), but for individual contributions from (b) temperature anomalies and (c) wind anomalies. Arrows indicate climatological-mean winds in (b) and wind anomalies in (c). Contour lines denote temperature anomalies (°C) in (b) and climatological-mean temperature in (c). Contour intervals are 0.4°C in (b) and 1°C in (c). These maps were generated with the GMT software (ver.5.4.4; https://www.generic-mapping-tools.org/download/).

(a) Zonal wind versus zonal temperature advection

(b) Zonal temperature gradient versus zonal temperature advection

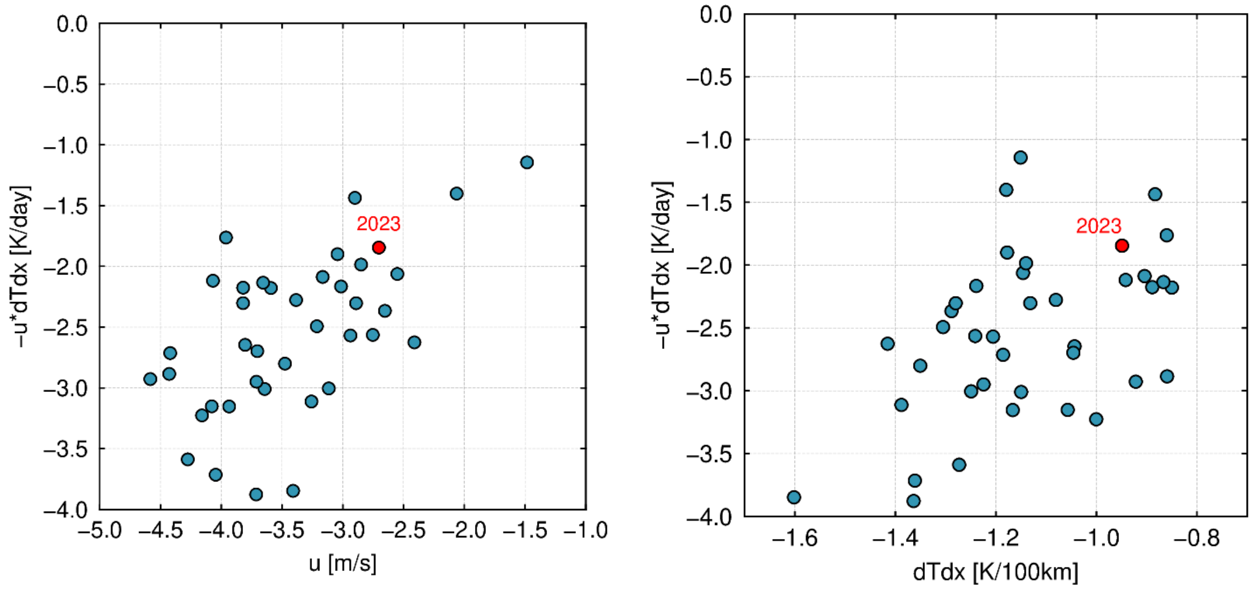

Supplementary Figure S10. Scatter plots of summer-mean (a) zonal wind (m/s) versus zonal temperature advection (K/day) and (b) zonal temperature gradient (K/100km) versus zonal temperature advection (K/day) at 975-hPa level averaged along the line [37.5°–41.25°N, 142.5°E] at 0600UTC (1500 local time), based on conditional sampling only when the surface zonal wind component was easterly. Red dots correspond to 2023 summer.

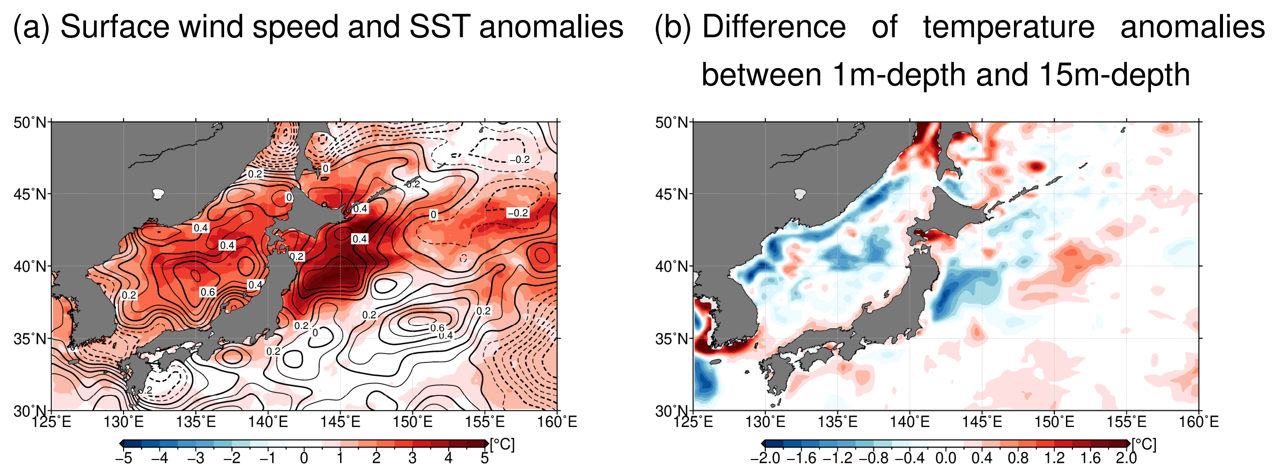

Supplementary Figure S11. (a) Anomalies of surface wind speed (m/s; contour) and SST (°C; colour) in the summer of 2023, where the surface wind speed is evaluated with 6-hourly wind field not a summertime mean field. (b) Difference of ocean temperature anomalies (°C) between 1m-depth and 15m-depth. These maps were generated with the GMT software (ver.5.4.4; https://www.generic-mapping-tools.org/download/).
